# Supplementary material for: Children with an Anorectal Malformation Going to Primary School: The Parent’s Perspective
Source: Children (Basel). 2023 May 24;10(6):924. doi: 10.3390/children10060924 (PMC10297550; doi:10.3390/children10060924)
Supplement: Supplementary file 1 [file children-10-00924-s001.zip › children-2388499-supplementary.pdf]

Supplementary Material File S1. Schoolproject questionnaire.

In advance

1. Do you, parent and/or legal representative of a child with an ARM, agree that by completing this questionnaire, this data will be used anonymously and irreducibly for scientific research and publication?
   a. Yes
   b. No (end of questionnaire)

General questions

1. What age is your child (in years)?
   …
2. How many schools did you visit before making your choice:
   a. 1
   b. 2
   c. 3
   d. 4
   e. 5 or more
3. During your search, were there any schools that made demands regarding your child's potty training (urination and defecation)?
   a. Yes
   b. No
4. Have you encountered any difficulties regarding enrolling your child in an elementary school?
   a. Yes
      i. Which?
         …
   b. No
5. What is the best description of your child’s school?
   a. < 100 children

  b. 100 to 500 children
  c. > 500 children

<u>Questions on experiences towards school</u>

1. Does your child have any negative experiences regarding urination or defecation at the current primary school?
   a. Yes
      i. Which?
         …
   b. No
2. Does your child have any positive experiences regarding urination or defecation at the current primary school?
   a. Yes
      i. Which?
         …
   b. No
3. What are the toilet facilities like at your child's current elementary school?
   …
4. How many toilets are there available for your child in school (number)?
   …
5. Are these toilets generally clean?
   a. Yes
   b. No
6. Are these toilets easily accessible and spacious?
   a. Yes
   b. No
7. Is there a toilet for children with disabilities available in the school?
   a. Yes
      i. Is your child allowed to use this toilet?
         1. Yes
            a. Is this toilet generally clean?

i. Yes

ii. No

b. Is this toilet easily accessible and spacious?

i. Yes

ii. No

2. No

b. No

8. Does your child use the toilet in school?

a. Yes

b. No

i. Why not?

…

9. Are there any specific provisions and/or arrangements made for your child regarding toileting at the current elementary school?

a. Yes

i. Were these arrangements already in place at the school?

1. Yes

2. No

ii. Has the school requested these arrangements?

1. Yes

2. No

iii. How were these arrangements established?

…

b. No

10. Does your child receive help at school?

a. Yes

i. For what?

…

ii. By whom?

…

b. No

11. Has your child switched schools before finishing primary school?
    a. Yes
        i. How many times?

        …
    b. No
12. Do you have any additional comments/suggestions/comments/questions or would you like to comment on this topic?
    a. Yes
        i. Room for questions

        …
    b. No (end of questionnaire)
